# Editorial: Our Canine Connection: The History, Benefits and Future of Human-Dog Interactions

**DOI:** 10.3389/fvets.2021.784491

**Published:** 2021-11-04

**Authors:** Eric G. Strauss, Sandra McCune, Evan MacLean, Aubrey Fine

**Affiliations:** ^1^The Center for Urban Resilience, Loyola Marymount University, Los Angeles, CA, United States; ^2^School of Psychology, School of Life Sciences, University of Lincoln, Lincoln, United Kingdom; ^3^Animal Matters Consultancy Ltd., Stamford, United Kingdom; ^4^Arizona Canine Cognition Center, School of Anthropology, University of Arizona, Tucson, AZ, United States; ^5^Department of Psychology, University of Arizona, Tucson, AZ, United States; ^6^College of Veterinary Medicine, University of Arizona, Tucson, AZ, United States; ^7^Cognitive Science Program, University of Arizona, Tucson, AZ, United States; ^8^California State Polytechnic University, Pomona, CA, United States

**Keywords:** human-animal bond, Annenberg PetSpace, canine domestication, working dogs, canine connection introduction

We are current witness to profound changes in the demography and social behavior of humans that includes paradigm shifts in science, religion and philosophy. Coupled with the profound impacts of plant and animal domestication, the modern forces of urbanization and technological transformation have created complex social communities yoked to the ecology and behavior of domestic animals whose roles were likely unimagined only a century ago. Scholars refer to the changing place of humanity on the planet and have named the current epoch the Anthropocene, but it would not have been possible without agricultural and animal domestication that dates back to antiquity. Our efforts to better understand the lives of working domestic animals have great potential to enhance the processes through which these animals are bred, selected, trained, and cared for, resulting in a more benevolent ecosystem for this mutual relationship. In this series, we present a suite of papers that aim to sharpen our understanding of the origins, behavior, cognition and welfare of the domestic dog, and apply these considerations to their working roles in society. The goal of this exercise is to provide a review of the science to date and suggest new frameworks for future use-inspired research in the canine sciences.

The genesis of this project was made possible and funded by the Annenberg PetSpace Foundation through the establishment of a Leadership Institute (https://www.annenbergpetspace.org/about/leadership). The Institute seeks to foster and support the understanding of the human-animal bond through research and program implementation. Fellowships are awarded by the Foundation in order to bring together experts with diverse backgrounds for intellectual exchange and visioning for future research. The first two rounds of the Fellowship Program focused on canine research and its applications. Each round included a multi-day retreat which served as an intellectual salon, with an open agenda for the exchange of ideas. The first retreat was held in 2017 (https://www.annenbergpetspace.org/about/leadership) and spanned a universe of expertise from history, genetics and evolution, to behavior, welfare and human social dynamics. Emerging from this effort was a single multi-author paper (1) that argued for carving out a space for canine science and the domestic dog in the context of tackling the declining environmental conditions of the globe. Titled *Humanity's Best Friend*, the team of authors considered the role of domestication and the complex relationship that we have with domestic dogs as tools to better achieve Sustainable Development Goals (SDG) as outlined by the United Nations.

Building on the success of this project, the Annenberg PetSpace Foundation hosted a second round of Leadership Fellows in 2020 (https://www.annenbergpetspace.org/about/leadership). The strategy employed at this retreat was to narrow the focus to the specific dimensions of working dogs, human animal interaction (HAI) research and implications for the understanding of domestication as a tool for both improving human health outcomes and those of our canine companions. What follows is the suite of 12 papers authored by select members of both retreats and their colleagues. The papers are organized into five broad categories, which we explore below.

## Evolution and Behavior (Three Papers)

Three papers address the issues of dog domestication, overall ecological character and genetics. Serpell tackles the controversial and dynamic story of the domestication of dogs from their wolf ancestors. Serpell compares the evidence for two predominant origin stories. The first suggests a commensal relationship of wolves exploiting food scraps from humans that gradually gave rise to a full-blown mutualism of collaborative hunting, resource protection and social companionship. The second model, which Serpell favors, suggests an active role for Paleolithic peoples who actively captured wolf pups as a form of pet-keeping, otherwise known as the cross-species adoption hypothesis. Serpell suggests that the ecologies of both species make such a phenomenon quite plausible.

Wynne picks up the domestication saga much later in time and explores the complex relationships humans have had with domestic dogs in recent history. Unlike theories that suggest that dogs are more compliant than wolves and therefore more likely to be domesticated, Wynne argues that the foraging nature of dogs facilitates a strong independence, unbounded by the complex social order of wolves. Along with this set of traits comes the ability of humans and dogs to interact across species boundaries, with Wynne characterizing the nature of this relationship as one of “super-dominance,” with allusion to Tinbergen's ethological concept of supernormal stimuli. The third contribution to this set of papers comes from the lab of Elinor Karlsson and is led by Chen et al. They argue for the implementation of modern tools of genomics to better understand the nature of dogs in general and specifically genetic potential for diverse working applications. They argue for the application of statistical genomic approaches as a way to revolutionize the field of working dogs leading to a much higher degree of success in selection and training of puppies. Featured is a step-by-step guide for breeders to implement estimated breeding values in their programs through genotyping and DNA sequencing.

## Training (2 Papers)

Two papers address issues regarding the training and selection of dogs for working roles. Hall et al. leads a team that reviews current practices in working dog training concluding that many of the techniques used today predate major advances in our understanding of dog cognition, many of which have occurred in the last two decades. The authors identify specific areas in which working dog training should be modernized for the Twenty-first century, and identify key research questions which have yet to be explored.

Bray et al. collaborates with a team to identify practices through which the selection and performance of working dogs can be optimized. They point out that while dogs provide very complex services in their roles as working animals, the processes by which they are selected and trained have numerous points of failure, which collectively are expensive and burdensome to trainers and stakeholders, and have potential negative implications for dog welfare and human health The authors stress the need to develop new selection criteria at the level of the individual candidate animal and to enhance breeding and rearing practices at the population level, in order to best serve working dogs and the humans they assist.

## Human Health (3 Papers)

Three papers explore the positive effects that working and companion dogs have on human health, with an emphasis on the impact of these relationships on the dogs as well. McCune and Promislow consider the arc of aging in humans and dogs, which differ considerably due to their variation in lifespans. This apparent disequilibrium actually provides a myriad of opportunities for a deeper understanding of the aging process and the mutual companionship opportunities that exist because of it. Using the World Health Organization's *Healthy Aging Initiative*, the authors explore the research on aging of both dogs and humans and how we can better understand and support this process through innovative research and program development.

Gee et al., along with Kerri Rodriguez use a biopsychosocial framework to contextualize current research on the contribution of human-animal interactions to human wellbeing. The authors also focus on a wide range of social contexts in which dogs have been involved in adjunct or complementary therapy to a diverse community of people in need. The authors provide a framework for analysis and help set an agenda for further research in this transdisciplinary field.

Finally, Barker and Gee consider the state of knowledge in the use of therapy dogs in formal hospital settings. The goal here is to maximize the benefit to human patients while also protecting the physical and mental health of the dogs. As they note, this places human medicine and the veterinary sciences in a collaborative partnership that currently lacks the appropriate understanding and policies to ensure success in all dimensions. The paper presents a clear rationale for canine-assisted interventions in a hospital setting and explores the challenges presented by these novel therapies. A two-decade program in a medical center is used as an exemplar along with a thorough analysis of the research in order to build the case for future expansion of these practices.

## Animal Welfare (2 Papers)

Two of the papers in this series deal directly with the welfare of working dogs and also the consideration of the world from the dog's perspective. Cobb et al. review papers on working dogs from the last decade (2011–2020) with an emphasis on human interaction, ethics and the five domains of animal welfare. As the awareness of, and research on animal welfare has increased, the authors use this growing body of research to evaluate past practices and current challenges for developing a sustainable and responsible model in which working dogs are embraced as co-workers.

Horowitz evaluates dog-human interaction under the umbrella of HAI research from the perspective of the welfare of the dog as the “silent partner.” In reviewing the literature, she poses fundamental questions about the appropriateness of dogs in research, the impact on the species and individuals, even developing a model for a dog's agency in research. She poses the idea that dogs should have a system of providing “consent” for participation, similar to what we expect from human research studies.

## Improving Research and Looking Forward (2 Papers)

Two of the papers have a focus on research methodologies and future challenges and opportunities of domestic canine science. Rodriguez et al. seek to improve the quality of future research by looking at the strengths and weaknesses of current methodologies. They investigate ways in which experimental design can be improved in order to sharpen the hypotheses that are being investigated and strengthen the conclusions that can be drawn from this work. These challenges are particularly acute with a species as cosmopolitan as the domestic dog. The onus on improving the research is an issue that has dogged the wild canid research community as well, especially with respect to managing coyotes (2). The convergence of concern in domestic and wild canid research suggests supplying a new standard to the research effort can amplify the effectiveness of the work and reduce the risk of implementing weak inferences from the findings.

The final paper from the MacLean et al. serves as a synthesis and prospectus that unifies the suite of papers and looks toward the future of use-inspired research in the field of anthrozoology. Focusing on a range of topics, relevant to stakeholders, funders and the research community, the authors delineate a path forward for this interdisciplinary work. Drawing on similar disciplines that investigate human-dominated natural systems and synanthropic species, such as urban ecology, we argue that HAI can have profound implications on human wellbeing and that of our canine partners. The collection of articles will provide a comprehensive synthesis of the current trends within the field. We hope you enjoy the series and find it relevant to your work and a catalyst to your aspirations.

## Author Contributions

All authors listed have made a substantial, direct and intellectual contribution to the work, and approved it for publication.

## Conflict of Interest

SM is a paid consultant, paid for by Annenberg PetSpace to lead the development of this special topic, the manuscripts of which, came from two workshops which they sponsored and he was employed by company Animal Matters Consultancy Ltd. And he is a paid consultant on the Research Advisory Board for the Waltham Centre for Pet Nutrition, Mars UK. The remaining authors declare that the research was conducted in the absence of any commercial or financial relationships that could be construed as a potential conflict of interest.

## Publisher's Note

All claims expressed in this article are solely those of the authors and do not necessarily represent those of their affiliated organizations, or those of the publisher, the editors and the reviewers. Any product that may be evaluated in this article, or claim that may be made by its manufacturer, is not guaranteed or endorsed by the publisher.
